# Case Report: A Novel *LAMP2* Splice-Altering Mutation Causes Cardiac-Only Danon Disease

**DOI:** 10.3389/fcvm.2021.763240

**Published:** 2021-11-25

**Authors:** Zongzhe Li, Fei Ma, Rui Li, Zhichao Xiao, Hesong Zeng, Dao Wen Wang

**Affiliations:** ^1^Division of Cardiology, Department of Internal Medicine and Genetic Diagnosis Center, Tongji Hospital, Tongji Medical College, Huazhong University of Science and Technology, Wuhan, China; ^2^Hubei Key Laboratory of Genetics and Molecular Mechanisms of Cardiological Disorders, Huazhong University of Science and Technology, Wuhan, China

**Keywords:** Danon disease, *LAMP2*, splicing mutation, targeted sequencing, genetic diagnosis

## Abstract

Danon disease (DD) is a rare glycogen storage lysosomal disorder caused by mutations in the *LAMP2* gene. Patients with DD are usually characterized clinically by severe multisystem syndromes. We describe a specific family with a novel pathogenic splice-altering mutation in the *LAMP2* gene (c.741+2T>C) with cardiac-only symptoms (frequent ventricular tachycardia, intraventricular block, and hypertrophic cardiomyopathy). Minigene assays were used to evaluate the consequence of the splice-site mutation in the *LAMP2* gene. The results showed that the c.741+2T>C mutation led to extra 6-bp preservation of intron 5 at the junction between exons 5 and 6 during transcriptional processing of the mRNA, which creates a stop codon and truncated the LAMP2 protein to 248-amino-acid residues. The mutant LAMP2 protein was predicted to have a conformational change, lacks the important transmembrane domain, and subsequent protein destabilization.

## Introduction

Danon disease (DD), with Online Mendelian Inheritance in Man (OMIM) No. 300257, is a rare X-linked dominant disorder caused by pathogenic mutations in the lysosomal-associated membrane protein 2 (*LAMP2*) gene (1–3). The *LAMP2* protein is essential for the protection, maintenance, and adhesion of the lysosome, therefore is integral to cellular autophagy (2). The *LAMP2* mutations typically lead to multisystem glycogen-storage lysosomal disease but can also present as primary cardiomyopathy with cardiac-only symptoms, which is not frequent in the literature (1, 4). The main clinical manifestations of patients with DD are usually multisystem involved: hypertrophic cardiomyopathy, myopathy, and intellectual disability (4–7). Male patients usually develop the condition earlier than female patients and suffered from more severe symptoms (1, 6). The relationship between specific genotypes and different phenotypes is not yet clear.

We presented a family with a novel splice-altering mutation (*LAMP2* c.741+2T>C) in 3'-splice site of exon 5 with cardiac-only symptoms (frequent ventricular tachycardia, intraventricular block, and hypertrophic cardiomyopathy).

## Materials and Methods

### Targeted Sequencing

The peripheral blood samples of the patient and his relatives were obtained. The genomic DNA of each participant was extracted using the QIAamp DNA Mini Kit (Qiagen, Germany) by following standard protocols. The isolated DNA was evaluated using electrophoresis to make sure without degradation or RNA pollution.

To rapidly discover the genetic cause of patients with malignant arrhythmia and cardiomyopathies, a “cardiomyopathy-ion channelopathy” panel, including 142 causal and candidate genes, was designed. The panel included full coding regions (542.22 kb) with at least 5-bp flanking regulatory sequences of causal and candidate genes for different hereditary cardiomyopathies and hereditary ion channelopathies. Amplicon-based libraries were constructed and were performed high-depth targeted semiconductor next-generation sequencing on an Ion Torrent Personal Genome Machine (PGM) (Life Technologies, USA) following the standard protocol (8). The following data filtration strategy is as we previously described (8).

### Sanger Sequencing

The identified pathogenic mutations and low coverage regions were further Sanger sequenced by using specific primers on an Applied Biosystems 3500xl sequencer (Applied Biosystems, USA). After validated in the patient, the potential pathogenic mutations were also Sanger sequenced in his relatives and extra 800 unrelated Chinese healthy controls.

### Copy Number Variations (CNVs) Analysis

We performed copy number variations (CNV) analysis through the targeted sequencing data using CNV workflow on Ion Reporter^TM^ software (Life Technologies, USA) as we previously described (9). All identified potential pathogenic CNVs were validated by quantitative real-time PCR analysis of gDNA.

### Minigene Assays

We designed a pair of minigene plasmids (wild-type and mutant) for the *LAMP2* gene 3' -splice site mutation (c.741+2T>C). The wild-type minigene construct was produced by cloning into the exon trap vector PET01 (MoBiTEc, Germany). The sequence of exon 5 of the *LAMP2* gene with partial intron 4 and 5 (1,396 bp) was amplified and inserted into the minigene plasmid (LAMP2-PET01) (**Figure 2A**). The target sequence was amplified from the genomic DNA of the patient or his father using the following specific primer pair: forward 5'-taggccccaggatagctcgagGTATCAGAGGCAGGCAAAGTTC-3' and reverse 5'-tgctctatggggtccggatccCTGATCCACTGATGGCAAATAGA-3'.

Human embryonic kidney (HEK 293) cell lines were cultured in DulbeccoŠs Modified EagleŠs Medium (DMEM) supplemented with 10% fetal bovine serum (Thermo Fisher, USA). Cells were grown at 37°C with 5% carbon dioxide (CO_2_). Cell transfection was performed using Lipofectamine 2000 (Invitrogen, USA) according to the protocol of the manufacturer.

### RNA Extraction, cDNA Synthesis, and Splice Site Analysis

Human embryonic kidney 293 cells were transfected in triplicates and total RNA was extracted after 48 h using a RNeasy Mini kit (Qiagen, Germany). The extracted RNA samples were treated with 5 U/μl DNase I (Takara, Japan) and the RNA quality was examined with both agarose gel (UltraPure, Invitrogen, USA) electrophoresis and a NanoDrop 2000 spectrophotometer (Thermo Fisher Scientific, USA). RT-PCR was performed with a Primer Script™ RT Reagent kit (Takara, Japan) using 1 μg total RNA.

To specifically amplify the target spliced cDNA products derived from the expressed minigene, we designed a pair of primers (433 bp): forward 5-GATCGATCCGCTTCCTGCCCC-3' and reverse 5'-TTCTGCCGGGCCACCTCCAG-3'. The forward and reverse primers were located in exon SD and exon SA, respectively. Subsequently, the PCR products were visualized by electrophoresis on a 2% agarose gel and were confirmed by Sanger sequencing.

### Protein Structural Modeling

To investigate the consequence of the identified mutation, the protein structural modeling source code for the AlphaFold model, trained weights, and inference script were available under an open-source license using AlphaFold Protein Structure Database (https://github.com/deepmind/alphafold) (DeepMind Technologies, USA) and figures were prepared with UCSF ChimeraX (RBVI, USA) (10).

## Results

### Clinical Phenotypes

**Figure 1:**
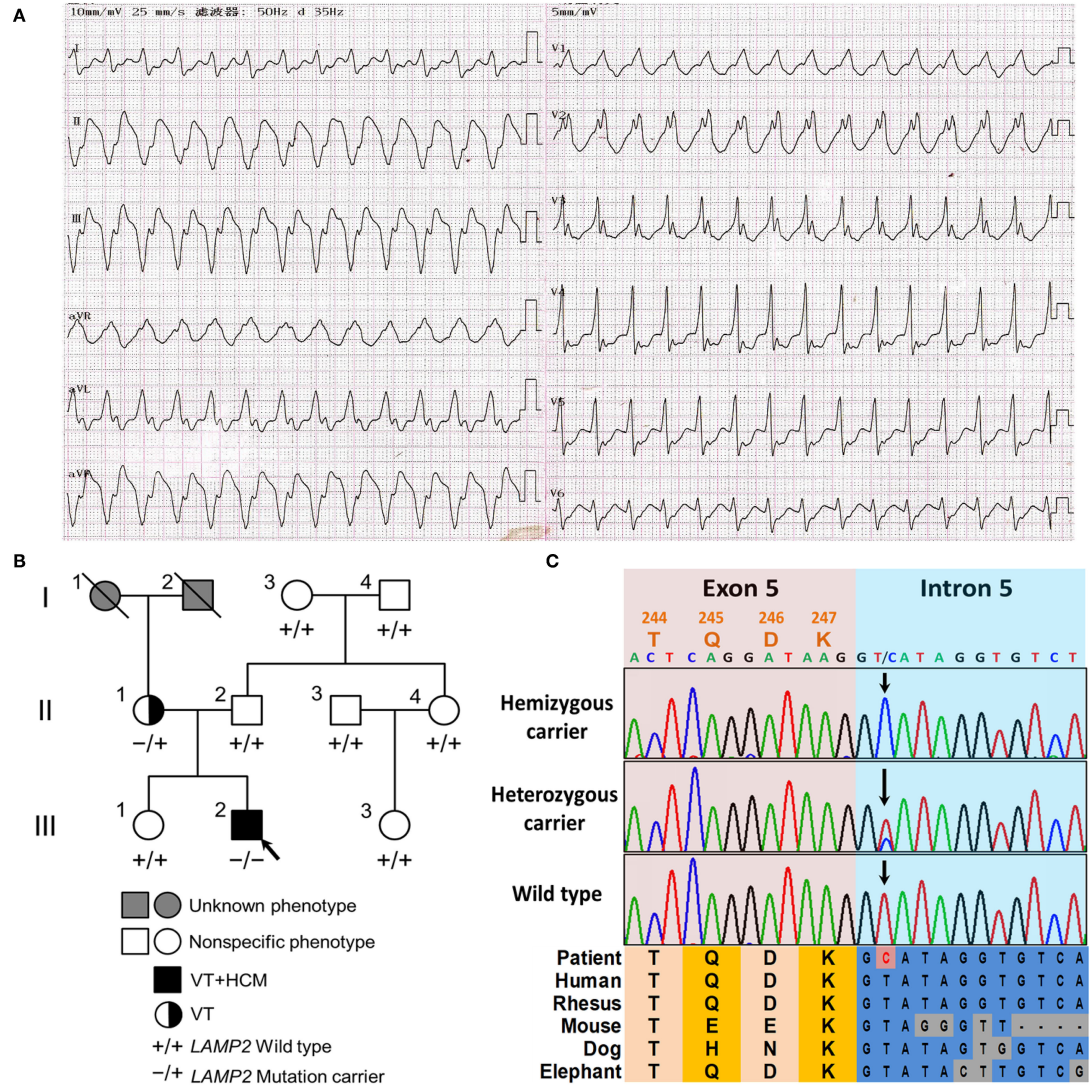
The clinical and genetic characteristics of the pedigree. **(A)** The 12-lead electrocardiogram (ECG) of the proband revealed ventricular tachycardia and intraventricular block. **(B)** The family tree of the cardiac-only DD pedigree is included in this report. Male and female are indicated by squares and circles, respectively. The black filled symbol represents the clinical affected individual. The gray-filled symbol represents the phenotype unknown individual. The arrow shows the proband. –/+ represents heterozygous *LAMP2* c.741+2T>C variant. –/– represents hemizygous *LAMP2* c.741+2T>C variant. +/+ represents a wild type. **(C)** The Sanger sequencing of the family. Colored blocks show the evolutionary conservation of the cluster across multiple species.

A 19-year-old normally developing man was referred to our hospital with a 3-month history of paroxysmal palpitations and dyspnea after exercise. The patient was mentally developing normally. The patient had normal bilateral muscle strength in the physical examination. The electromyography was normal. The patient denied any history of myalgia or gait disturbance. The echocardiography revealed hypertrophic cardiomyopathy in left ventricular end-diastolic dimension (50 mm), left atrium (34 mm), interventricular septum end-diastolic thickness (20 mm), and posterior wall end-diastolic thickness (18 mm), with a left ventricular ejection fraction of 50%. The 12-lead ECG and 24 h Holter ECG monitoring of the patient revealed paroxysmal ventricular tachycardia and intraventricular block (Figure 1A). In laboratory tests, his NT-proBNP, TnI, CK, lactic acid, liver and kidney function, levels of serum electrolytes, blood glucose, and lipids were all in the normal range. The patient had no family history of sudden cardiac death.

The 42-year-old mother of the patient had no other clinical manifestation besides occasional palpitation after exercise. The mother was also well-developed and mentally developing normally. The mother was also diagnosed with paroxysmal ventricular tachycardia by 24 h Holter ECG monitoring, however, the echocardiography did not show obvious anomalies. The blood examinations (NT-proBNP, TnI, CK, lactic acid, liver and kidney function, levels of serum electrolytes, blood glucose, and lipids) of the mother were all in the normal range. The rest of his relatives did not show obvious anomalies during physical or laboratory examinations (Figure 1B). In the follow-up of 2 years, all the family members did not develop neurological or muscular symptoms.

### Genetics Analysis

After filtering and Sanger sequencing validation, we identified a pathogenic hemizygous 3' splice site mutation, c.741+2T>C, in intron 5 of the *LAMP2* gene (Figure 1C), according to the ACMG guideline (11). No other potential pathogenic variants or CNVs were filtered in the coding regions and flanking regulated regions of known hypertrophic cardiomyopathy or ion channelopathy-related genes. The mutation was also identified in the mother (heterozygous) but was not identified in the rest relatives or the extra 800 unrelated Chinese healthy controls (Figure 1). The mutation was absent in all public databases. Furthermore, the mutation was in an evolutionary highly conserved region across multiple species (Figure 1C).

### Splicing Analysis

**Figure 2:**
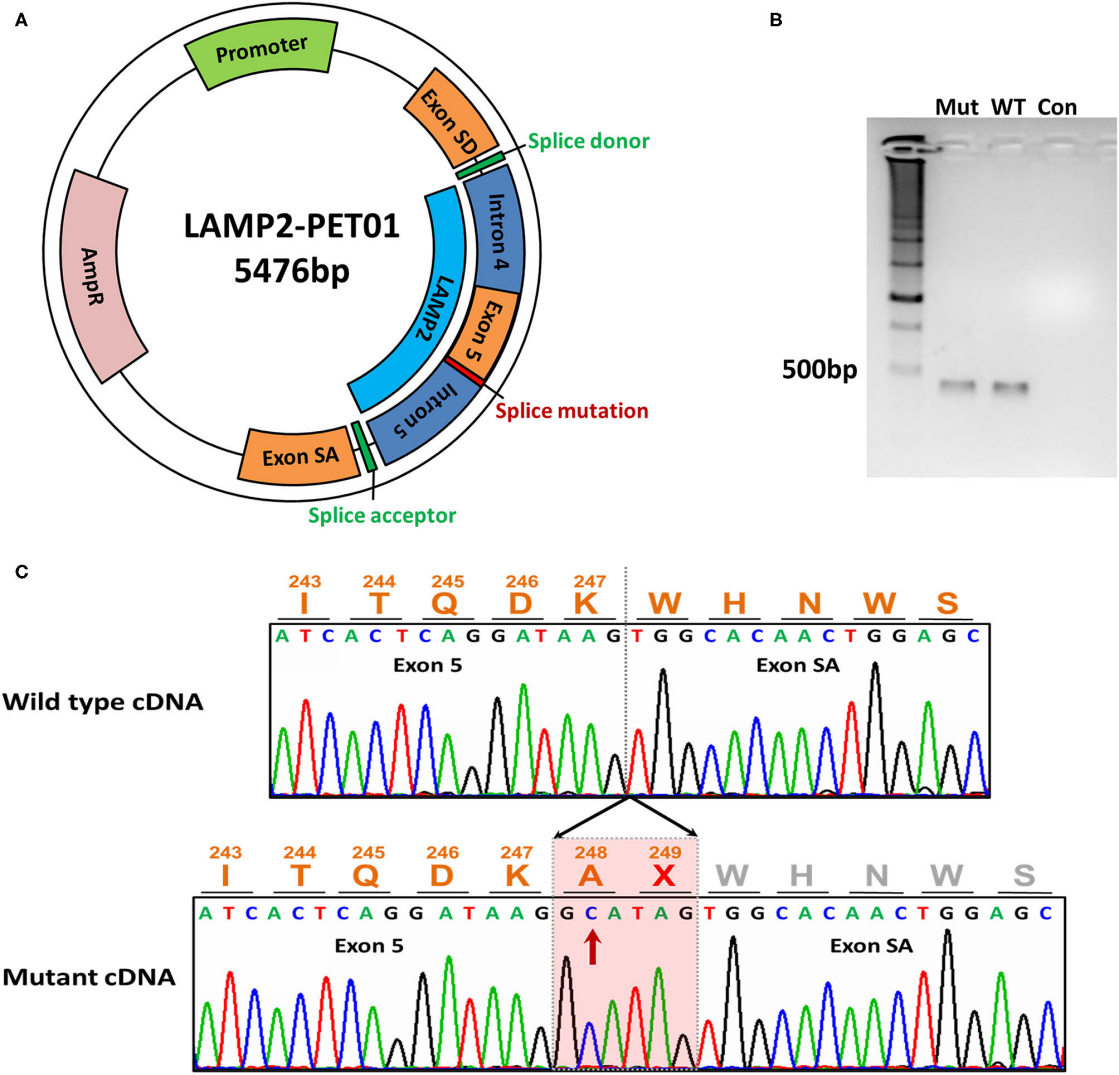
The minigene assay of the mutation. **(A)** The construction of the LAMP2-PET01 minigene plasmid. **(B)** Electrophoresis of the cDNA products. Total RNA was extracted after 48 h the minigene plasmid was transfected and reverse transcribed into cDNA. Mut represents cDNA from mutant plasmid treated. WT represents cDNA from wild-type plasmid treated. Con, represents blank control without any cDNA. **(C)** Sanger sequencing of the cDNA products. Sequencing identified extra 6-bp preservation of intron 5 at the junction between exons 5 and exon SA during transcriptional processing of the mRNA, which creates a stop codon.

In order to evaluate the bioinformatic predictions, we further investigated the effects of the identified 3' splice site mutation (c.741+2T>C) in the *LAMP2* gene by minigene assays. The results showed that the mutation disrupted the splice donor site (GT) in intron 5. We compared the RT–PCR products of wild-type and mutant mRNA by electrophoresis and found the mutant cDNA was a little larger than the wild-type cDNA (Figure 2B).

By Sanger sequencing, we identified a 6-bp insertion at the junction of exons 5 and 6 in the mutant cDNA. The inserted nucleotides had the same sequence as the 5'-end of intron 5, which implied the preservation of intron 5 at the junction between exons 5 and 6 during transcriptional processing of the mRNA (Figure 2C).

### Protein Structural Modeling

**Figure 3:**
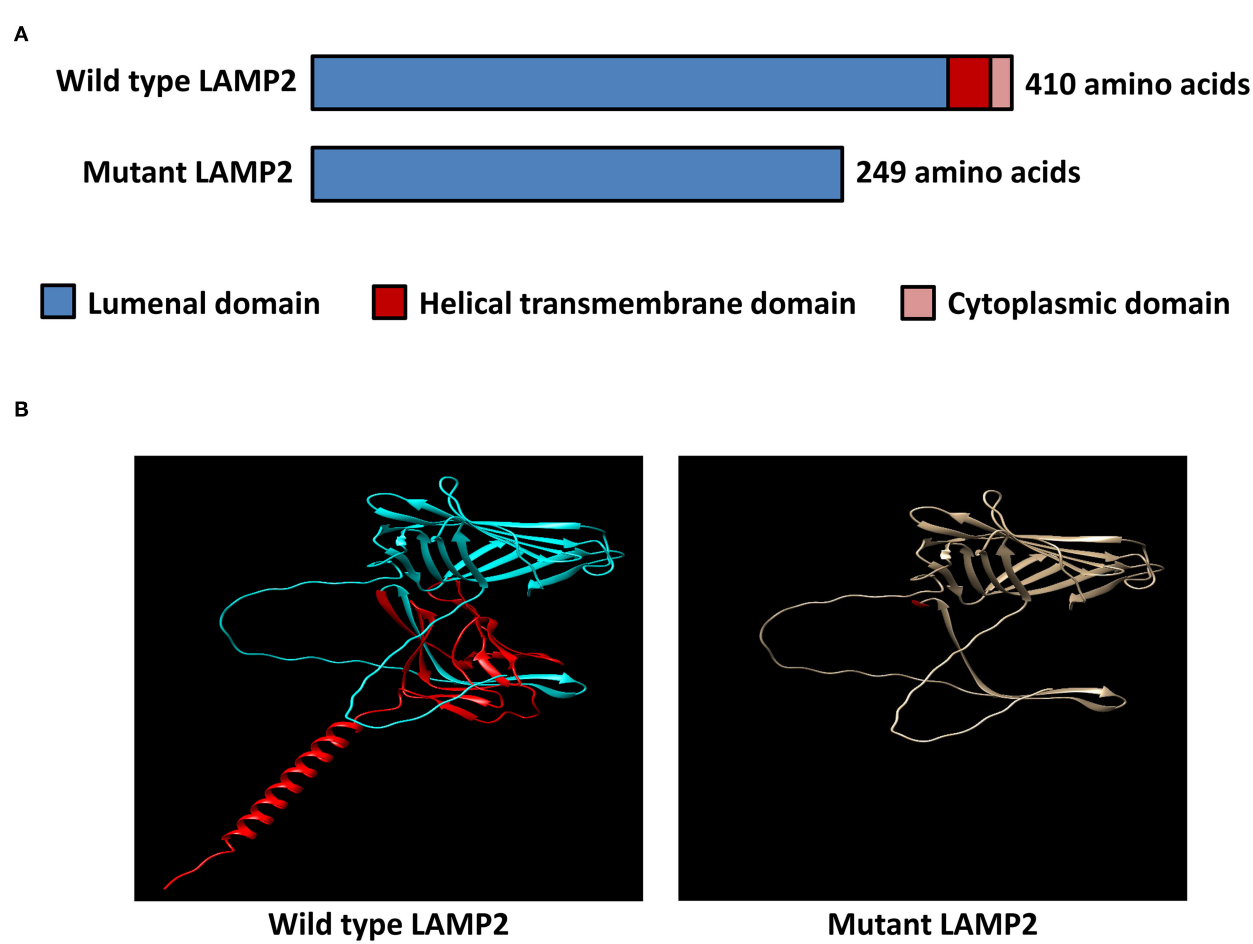
*In-silico* structural modeling of the identified mutation. **(A)** Schematic diagram of the wild-type LAMP2 protein and the mutant truncated LAMP2 protein. **(B)** Protein structural modeling of the wild-type and mutant LAMP2 protein.

The inserted sequence encoded an in-frame stop codon, which predicted a premature termination of the nascent polypeptide (Figure 2C). The mutation truncated the LAMP2 protein to 248-amino-acid residues, which lacks the transmembrane domain and the cytoplasmic domain, therefore, fail to function properly (Figure 3).

## Discussion

In this study, the proband was diagnosed with cardiac-only DD based on genetic testing, functional experiments, and clinical examinations. By targeted high-depth semiconductor sequencing and following functional analysis, we presented a genetic report linking a novel splice-altering *LAMP2* mutation to rare cardiac-only DD syndrome and revealed the transcriptional pathogenesis of this mutation.

The *LAMP2* c.741+2T>C mutation was identified in the proband (hemizygous) and his mother (heterozygous) but was absent in other healthy relatives, which implied a co-segregation in the pedigree (Figure 1B). The mother harbored a wild-type allele and a mutant allele, therefore suffering a milder clinical symptom, only occasionally palpitation after exercise due to paroxysmal ventricular tachycardia, which was in accordance with previously reported (1, 6). The main symptoms of the two patients in the pedigree were based on arrhythmia and were lack of symptoms associated with skeletal myopathy and mental retardation, which is quite different from typical reported DD clinical features (1).

The mutation was neither in the Exome Aggregation Consortium (ExAC; http://exac.broadinstitute.org/) database, nor the extra 800 healthy controls. It was also absent in the ClinVar (https://www.ncbi.nlm.nih.gov/clinvar/) and HGMD (http://www.hgmd.cf.ac.uk/ac/search.php) databases. The mutation was predicted to be located in an evolutionary highly conservative locus (Figure 1C).

The minigene assays revealed that the novel 3'-splice site mutation (*LAMP2* c.741+2T>C) caused a 6-nucleotide (two codons) insertion in the mRNA between exon 5 and exon SA (Figure 2). Importantly, the second inserted codon was a stop codon, leading to the early termination of LAMP2 protein during translation (Figure 3). The mutant LAMP2 protein has only 248-amino-acids residues, therefore, lacks the important transmembrane domain and the cytoplasmic domain (Figure 3). In the previous studies, another pathogenic splice-altering mutation (*LAMP2* c.741+1G>A, rs1251075016) next to our mutation was reported (2, 12–14). The reported mutation was demonstrated to cause the same 6-bp mRNA preservation and protein-truncating consequences by using skeletal muscle biopsy samples of the patients with DD (2). However, the clinical characteristics of patients who harbored the reported nearby splice-altering mutation (rs1251075016) were in accordance with typical DD (hypertrophic cardiomyopathy, myopathy, and mental retardation), which is different from our patients (cardiac-only symptoms). The clinical differences between these two splice-altering mutations may be caused by detailed transcriptional modifications and need further investigation.

To date, 17 splice-altering mutations in the *LAMP2* gene are included in the ClinVar database. The vast majority of them were considered to be pathogenic mutations. However, the observed clinical phenotypes due to different splice-altering mutations were not identical. Some of these mutations cause symptoms involving only the heart (1, 15), while other mutations also cause clinical manifestations of multi-organ damage including cardiac hypertrophy, mental retardation, and musculoskeletal defects (2, 13, 14). Even the same disease-causing mutation can lead to different clinical phenotypes (1, 16). The clinical phenotypic spectrum of DD is complex, and the relationship between different genotypes and clinical phenotypes deserves further exploring. In conclusion, we identified a novel splice-altering mutation in a Chinese family with cardiac-only DD and revealed the transcriptional pathogenesis of this mutation. Our findings enriched the pathogenic spectrum of the *LAMP2* gene and the phenotype profile of DD. This report would facilitate future genetic diagnosis and genetic counseling.

## Data Availability Statement

The data presented in the study are deposited in the SRA repository, accession number SRR16526542.

## Ethics Statement

The studies involving human participants were reviewed and approved by the Ethics Committee of Tongji Hospital, Tongji Medical College, Huazhong University of Science and Technology. The patients/participants provided their written informed consent to participate in this study. Written informed consent was obtained from the relevant individual for the publication of any potentially identifiable images or data included in this article.

## Author Contributions

DW designed and supervised the study. ZL, FM, RL, and ZX participated in the data interpretation and analysis. ZL, ZX, and HZ reviewed clinical data and offered diagnoses. ZL wrote the manuscript. All authors read and approved the final manuscript.

## Funding

This work was supported by grants from the National Natural Science Foundation of China (81700413).

## Conflict of Interest

The authors declare that the research was conducted in the absence of any commercial or financial relationships that could be construed as a potential conflict of interest.

## Publisher's Note

All claims expressed in this article are solely those of the authors and do not necessarily represent those of their affiliated organizations, or those of the publisher, the editors and the reviewers. Any product that may be evaluated in this article, or claim that may be made by its manufacturer, is not guaranteed or endorsed by the publisher.
